# Noninvasive Recognition and Biomarkers of Early Allergic Asthma in Cats Using Multivariate Statistical Analysis of NMR Spectra of Exhaled Breath Condensate

**DOI:** 10.1371/journal.pone.0164394

**Published:** 2016-10-20

**Authors:** Yan G. Fulcher, Martial Fotso, Chee-Hoon Chang, Hans Rindt, Carol R. Reinero, Steven R. Van Doren

**Affiliations:** 1 Department of Biochemistry, 117 Schweitzer Hall, University of Missouri, Columbia, MO, 65211, United States of America; 2 Department of Veterinary Medicine and Surgery, College of Veterinary Medicine, University of Missouri, 900 East Campus Drive, Columbia, MO 65211, United States of America; Imperial College London, UNITED KINGDOM

## Abstract

Asthma is prevalent in children and cats, and needs means of noninvasive diagnosis. We sought to distinguish noninvasively the differences in 53 cats before and soon after induction of allergic asthma, using NMR spectra of exhaled breath condensate (EBC). Statistical pattern recognition was improved considerably by preprocessing the spectra with probabilistic quotient normalization and *glog* transformation. Classification of the 106 preprocessed spectra by principal component analysis and partial least squares with discriminant analysis (PLS-DA) appears to be impaired by variances unrelated to eosinophilic asthma. By filtering out confounding variances, orthogonal signal correction (OSC) PLS-DA greatly improved the separation of the healthy and early asthmatic states, attaining 94% specificity and 94% sensitivity in predictions. OSC enhancement of multi-level PLS-DA boosted the specificity of the prediction to 100%. OSC-PLS-DA of the normalized spectra suggest the most promising biomarkers of allergic asthma in cats to include increased acetone, metabolite(s) with overlapped NMR peaks near 5.8 ppm, and a hydroxyphenyl-containing metabolite, as well as decreased phthalate. Acetone is elevated in the EBC of 74% of the cats with early asthma. The noninvasive detection of early experimental asthma, biomarkers in EBC, and metabolic perturbation invite further investigation of the diagnostic potential in humans.

## Introduction

Asthma is characterized by airways with inflammatory infiltrates, hyper-responsiveness, remodeling, and limited airflow, i.e. shortness of breath [1]. Asthma can range from mild to life-threatening. Almost 10% of children in industrialized countries develop asthma [2]. This places a disproportionate burden on health care systems, with incremental costs estimated at $56 billion in the US in 2007 [3].

The domestic cat is the only animal species that spontaneously develops a syndrome of asthma which replicates the hallmark features of the human disease [4]. Feline asthma is itself a major veterinary concern that affects between 1 and 5% [5] of the 74 million pet cats in the U.S. The similarities between human and feline allergic asthma led to the establishment and characterization of a feline experimental model of allergic asthma that mimics the hallmark clinicopathologic features in humans [6–8]. Allergic asthma is experimentally induced in cats using the clinically relevant Bermuda grass allergen (BGA) as aeroallergen to induce airway eosinophilia, airway hyperresponsiveness and histologic evidence of airway remodeling [6]. Additionally, cats develop spontaneous clinical signs after allergen challenge (cough, wheeze and/or labored breathing on exhalation), BGA-specific IgE and a T helper 2 cytokine profile [6]. This model is robust and has been used to investigate a variety of relevant clinical therapies including inhibitors of neurogenic inflammation, antihistaminics / antiserotonergics, enantiomers of albuterol, allergen-specific immunotherapy, small molecule inhibitors, and adipose-derived mesenchymal stem cells, among others [9–14]. Since eosinophilic airway inflammation drives airway hyperresponsiveness and remodeling, it is the primary outcome measure evaluated in this model. Aeroallergen-induced asthma is utilized herein to develop a noninvasive diagnostic approach to feline asthma.

Current clinical diagnostic methods are imprecise, low in sensitivity, and frustrated by the overlapping symptoms of other lower airway disorders [15, 16]. Consequently, there has been a clear need for accurate and objective early detection of asthma, as well as for means of ongoing monitoring of asthma and its management [17–19]. The clearest indication of allergic asthma in cats, together with clinical signs, is ≥ 17% eosinophils in the cytology of bronchoalveolar lavage fluid (BALF) [20]. Several barriers have been encountered in the development of disease-specific biomarkers for asthma diagnosis: Standard clinical tests for asthma such as the cytology of BALF are accurate but invasive and impart some risk, making the approach unsuitable for serial sample collection from children and pet cats. NMR spectra of urine samples of children, interpreted using supervised statistics, showed high accuracy in separating stable, chronic asthma from health or from acute asthma [17]. In serum and urine, no abnormalities have thus far been found to be definitively diagnostic for asthma in cats however [16]. Although concentrations of metabolites in exhaled breath condensate (EBC) samples are much lower and harder to detect than in serum or urine, EBC collection is noninvasive and directly samples airway fluids and the lung microenvironment.

Metabolomics of body fluids, including EBC, often relies on measurements by mass spectrometry or NMR spectroscopy, as they are sensitive enough to detect unique spectral “fingerprints” of a wide variety of metabolites [21, 22]. NMR has the advantages of quick and quantitative measurement of body fluids, good resolution of spectral peaks, and no manipulation or destruction of samples [21]. NMR spectra of EBC interpreted statistically attained a high degree of discrimination of healthy from asthmatic children [18]. NMR assay of EBC from 79 asthma patients discriminated those with neutrophil-rich sputum or using inhaled corticosteroids with high accuracies of 79% and 85%, respectively [23]. This relied upon use of multiple regions of the NMR spectra in the statistical analyses [23]. Even higher discrimination of health from the mild asthma of a smaller number of patients was obtained by interpreting NMR spectra of EBC using supervised statistics, regardless of the EBC being collected at -5 or -27°C [24]. Gas chromatography–mass spectrometry (GC-MS) detection of volatile organic compounds in EBC with statistics classified asthma with an accuracy of 96% [25]. Statistical models from the same study successfully classified the patients with well-controlled asthma or eosinophilia or neutrophilia in sputum [25]. Our purposes described below have been to investigate procedures, performance, and possibilities for analyzing NMR-based “breathprints” for noninvasive “breathomics” of asthma [22, 24], using the feline model of allergic asthma.

Metabolomics studies have often proceeded by untargeted [21, 26] or targeted approaches [27, 28]. The former divides each NMR spectrum into segments called bins or buckets for investigation of the *proportions* that the bins contribute to the total of the statistical variances across the measurements compared [26]. The more recent targeted approach estimates *absolute concentrations* by comparison with a library of reference spectra of the individual metabolites found in the body fluid [27]. Both approaches proved highly effective in discriminating mild asthma from health from NMR spectra of EBC analyzed with supervised statistics [24]. The concentration-dependent targeted strategy proved to be an enhancement with tighter clustering of the patient groups [24]. However, the composition of EBC has been unclear. No libraries of spectra of NMR-observable compounds in EBC have appeared at this writing for estimating their concentrations, as evident from the latest software for targeted profiling [28]. Consequently, the established untargeted approach using spectral binning was adopted for the current study. The sparseness of EBC spectra and high resolution at 800 MHz moderate the concern of peak overlap in bins.

Choice of preprocessing steps is crucial and heavily influences the perceived importance of metabolites [29]. Normalization of spectra by their integral addresses the highly variable concentrations of specimens of urine and EBC, but can be skewed by strong signals to introduce artifacts [30, 31]. This systematic error can be overcome by calculating the most probable dilution factor using probabilistic quotient normalization (PQN) [31]. After suitable preprocessing, multivariate statistics are applied to find patterns associated with biological status [21, 26]. The popular unsupervised method of principal component analysis (PCA) simplifies or projects the many measured features (variables) in spectra down to fewer uncorrelated principal components (PCs) that suggest trends shared among measured variables in the spectra [26, 32–34]. Variations on partial least squares (PLS) are popular to predict the class or phenotype of the sample, after an initial phase of training the statistical model [26, 32]. PLS uses multiple linear regression to derive from the data matrix **X** (here, the NMR spectra) the significant components related to the categories or phenotype **Y** (here, the presence or absence of allergic asthma in the feline patients). This makes use of “latent variables” not only to represent **X**, which PCA also represents, but also to correlate **X** with **Y** [26, 32]. A function called discriminant analysis (DA) is typically applied to clarify separation between categories [26, 32].

However, PLS-DA can be hampered by variation unrelated to the categories of interest [32]. In order to overcome large variations between subjects or patients, multi-level PLS-DA was proposed for sets of data where each subject has a before and after condition (“crossover design”)[35], e.g., before and after asthma induction in this work. By separating the between-subject variation from within-subject variation, the within-subject variation accompanying disease or treatment can be analyzed by PLS-DA with potentially improved classification [35]. As an alternative to improve PLS-DA classification performance, orthogonal signal correction (OSC) can be applied to the data to remove the contributions of variances unrelated (orthogonal) to the class **Y**, and thereby improve categorization [36–38].

This work investigates the potential of untargeted metabolomics using NMR spectra of EBC, interpreted using multivariate statistics, to diagnose asthma noninvasively in cats. The study exploits the feline model of allergic asthma induced by Bermuda grass allergen [6] by comparing the EBC of cats in a state of health with a subsequent state of allergic asthma early after induction (six weeks after initial exposure to antigen). The choice of preprocessing with PQN normalization and *glog* transformation proved pivotal. Classification of health or early asthma among 106 samples using statistically validated OSC-PLS-DA or multi-level PLS-DA appears superior, with excellent sensitivity and specificity. OSC-PLS-DA points out the trends of increase in acetone, increase in two metabolites of unclear identity, and decrease of phthalate in EBC from cats with allergic asthma.

## Materials and Methods

### Animal Care

All animals were domestic shorthair, purpose-bred cats. 27 were from a commercial vendor (Liberty Research, Inc, Waverly, NY). The remainder were bred from a high-responder asthmatic cat colony (Comparative Internal Medicine Laboratory, University of Missouri, Columbia MO). There were 39 males and 14 females. The care of the 53 cats in the study followed the NIH Guide for the Care and Use of Laboratory Animals. The University of Missouri Animal Care and Use Committee approved the study design (ACUC protocols #6912 and 7891). A commercial mixture of kitten and adult maintenance dry kibble diet was fed *ad libitum*. The research cats were housed in groups in large runs, with a variety of enrichment toys (e.g, hanging hammocks, elevated platforms to climb, baby toys, toy mice, balls with bells, etc.). Additionally, the cats were socialized and monitored at least daily by members of the research team, in addition to being monitored by members of the Office of Laboratory Animal Care. None of the cats became ill or died during this study. All of the cats were adopted to private homes after the study.

### Induction of Allergic Asthma

In order to induce allergic asthma, on day 0, cats < 1 year of age were administered 12 μg of Bermuda grass allergen (BGA) in 10 mg of alum and 100 ng *Bordetella pertussis* toxin (both subcutaneously). On day 14, intranasal BGA (75 μg of BGA in 200 μL of phosphate-buffered saline) was given. On day 21, 12 μg of BGA in 10 mg alum was injected subcutaneously. On day 28, the formation of wheals on an intradermal test confirmed sensitization to BGA. For the next 2 weeks, intensive aerosol challenges were administered to all cats with BGA (500 μg of BGA in 4 mL of phosphate-buffered saline) over 10 min in awake and unrestrained cats in a sealed plastic chamber. A nebulizer (Acorn nebulizer, model 646, Devilbis Health Care, Somerset, PA) delivered an aerosol of the BGA solution at an air flow rate of 9.3 l /min. An air compressor (Easy Air 15, Precision Medical, Inc., Northampton, PA) supplied the compressed air flow at a pressure of 2.93 kg/cm^2^. At week 6, samples were collected 24 h after challenge with BGA aerosol (see below).

### Collection of Body Fluids and Evaluation of Airway Eosinophilia

In all cats, EBC and BALF were collected before (day 0) and after (week 6) sensitization to BGA. The EBC was collected non-invasively just prior to BALF collection by placing cats in a 25 L plexiglass chamber for 20 to 30 min according to a previously published design with modifications [39]. BALF was collected in a blind fashion under anesthesia using an 8 French red rubber catheter passed through an endotracheal tube according to a previously described protocol [40]. The percentage of eosinophils in each BALF modified Wright’s stained cytospin was determined by counting 200 nucleated cells, with an asthmatic phenotype defined as >17% eosinophils. The EBC and the supernatant of centrifuged BALF remaining were promptly stored at -80°C until further analysis. NMR spectra of the EBC were measured for use in training and evaluating statistical approaches and biomarkers.

### Collection of NMR Spectra of EBC

To maximize the sensitivity and resolution of NMR measurements of inherently dilute EBC specimens, we collected their spectra with a Bruker Avance III 800 MHz spectrometer with 5 mm TCI cryogenic probe. EBC samples were prepared with 7% D_2_O and 20 μM trimethylsilyl propanoic acid as 0 ppm reference standard. 1D ^1^H NMR spectra were collected using W5-WATERGATE water suppression [41] supplemented by presaturation of the water resonance at minimal power. 32,768 transients were normally averaged. After data collection, the ^1^H NMR free induction decays were zero filled to 32,768 points, apodized with 2 Hz exponential broadening to enhance sensitivity, and Fourier transformed into spectra spanning from -2 to 12 ppm, with correction of phases and baseline using Bruker Topspin 3.1.

### Spectral Preprocessing

About 30,000 points from 0.02 to 10 pm were retained for analyses, while the region around the suppressed water peak from 4.5 to 5.38 ppm was omitted. Preprocessing steps were conducted with Topspin and Origin. To accommodate the large variability in overall concentrations of biomolecules condensed in EBC, the amplitudes of the NMR spectra were normalized to the mean spectrum by probabilistic quotient normalization (PQN) [31]. The first step of the PQN was unit normalization of the integral of each spectrum to 1.0. The median quotient was calculated from the quotients of all the spectral positions (variables) to those of the mean spectrum [31]. Then all variables of each original spectrum were divided by the median quotient of that spectrum. The NMR spectra of EBC specimens were next binned into segments to accommodate slight inequities in NMR peak positions and line shapes as recommended [26]. (EBC spectra are sparse enough to avoid the greater overlap within buckets of spectra of serum and urine [24]. Collecting the spectra at 800 MHz resolved the overlap of peaks enough to improve the reliability of binning. Shortcomings of bins have been discussed [30]). Each spectrum was divided into 455 bins each 0.02 ppm (16 Hz) wide and was then scaled with either Pareto scaling or *glog* transformation for comparison. Pareto scaling used the following expression where ${\tilde{x}}_{mn}$ is the scaled peak height [29]:

$${\tilde{x}}_{mn}=\frac{{x\prime }_{mn}}{\sqrt{s_{n}}},\;where\;s_{n}=\sqrt{\frac{\sum_{m=1}^{M}{\left({x\prime }_{mn}-{\overset{´}{x}}_{n}\right)}^{2}}{m-1}}$$ (1)

Scaling by *glog* transformation instead proved much better at increasing the weighting of the smaller variances. It uses the relationship [42]:

$${\tilde{x}}_{mn}=\ln({x^{\prime }}_{mn}+\sqrt{{x^{\prime }}_{mn}^{2}+\lambda )}$$ (2)

where λis the *glog* transformation parameter. λwas obtained by dividing the same sample into eight replicates (aliquots). Each replicate was handled independently for the NMR analysis. The spectra of the replicates were preprocessed identically to ensure that the variances from the replicates arise solely from “technical” variations. In order to avoid the scaling effect caused by the transformation, the modified Jacobian of the *glog* function proposed [42] was applied. λwas then calibrated using the Maximum likelihood criterion and Nelder-Mead minimization algorithm in MATLAB, using the equation and script provided in ref [42]. Data were mean-centered at the outset of multivariate statistical analysis.

### Multivariate Statistical Analyses and Validation

PCA decomposes the spectra (data matrix **X**) into PCs, which are a linear combination of weighted variables observed in the spectra. The optimized weights generate a loading plot. Each point of the score plot represents a spectrum’s projection onto the PC. The PCs were computed using eigenvectors by singular value decomposition [43]. PCA was performed using the MATLAB Statistics Toolbox. (The R-script princomp is also suitable; see http://www.inside-r.org/r-doc/stats/princomp).

PLS generated components instead using multiple linear regression, not only to model the spectral data matrix **X**, but also to calculate the projection matrix that maximizes the covariance between **X** and the class matrix **Y** of “response variables” [21, 26] or asthmatic phenotypes. Discriminant analysis (DA) was then applied to seek separation between the categories [21, 26] of absence and presence of allergic asthma. PLS-DA was performed with MATLAB using libraries of H. Li available from http://www.mathworks.com/matlabcentral/fileexchange/47767-libpls-1-95-zip. (R script plsda is also suitable: http://www.inside-r.org/packages/cran/mixOmics/docs/plsda). The collection of EBC before and after allergic induction of asthma was exploited in order to evaluate the multilevel PLS-DA approach to this crossover design in the data [35]. Multilevel PLS-DA was implemented in MATLAB with M-files developed at the Univ. of Amsterdam [44] and available at: http://www.bdagroup.nl/content/Downloads/software/software.php

Preprocessing by filtering out one OSC component to remove the largest variance of **X** orthogonal to **Y** (again, presence or absence of asthma) [36–38] was evaluated as well. OSC-PLS-DA calculations used the R script at: https://gist.github.com/dgrapov/5166570. To avoid overfitting, leave-one-out cross-validation was performed to select the optimal number of the components for use in model prediction. The prediction power of the statistical modeling was evaluated and validated by intensive Monte Carlo cross-validation and permutation testing.

### Identification of Pertinent Metabolites and Implications

For identifying dilute small molecules trapped in EBC, high sensitivity was obtained using the 800 MHZ NMR system with cryoprobe and overnight signal averaging of sensitivity-enhanced ^13^C heteronuclear single quantum coherence (HSQC) [45–47]. For broad bandwidth, the HSQC used chirp adiabatic inversion and refocusing pulses [48]. Reference NMR spectra were found at the Human Metabolome Database (HMDB) [49, 50] and Madison-Qingdao Metabolomics Consortium Database (MMCD) [51]. The database and server named Complex Mixture Analysis by NMR (COLMAR) ^13^C-^1^H HSQC [52] was used in semi-automated comparisons of these databases’ reference spectra with the natural abundance ^13^C HSQC in order to identify multiple carboxylic acids detected.

Statistical total correlation spectroscopy (STOCSY) was proposed for identifying biomarkers by calculating the correlation matrix of the NMR data sets [53]. A STOCSY correlation map was prepared from the 106 NMR spectra once preprocessed, segmented into 0.02 ppm bins, and *glog*-transformed. A ^1^H-^1^H correlation matrix (455X455 points) was generated from these preprocessed spectra. Covariance with a correlation coefficient r ≥ 0.7 was considered as significant and plotted in the STOCSY correlation map.

Variable Importance in Projection (VIP) from the OSC-PLS-DA and OSC-corrected multi-level analyses was calculated in order to evaluate the diagnostic significance of each spectral bin. Spectral bins with VIP value larger than 1.0 are considered to discriminate between groups [54]. Potential pathways affected by induction of the early stage of allergic asthma were anticipated by submitting the biomarker candidates to the MetaboAnalyst 3.0 server [55] at: http://www.metaboanalyst.ca/faces/ModuleView.xhtml

## Results

EBC and BALF specimens were collected from a research cohort of 53 cats when healthy, and six weeks after beginning the protocol of exposing them to Bermuda grass antigen in order to induce allergic asthma. The most trusted evidence of allergic asthma in cats is the elevation of eosinophils above 17% in BALF [20]. Before sensitization, the eosinophils averaged 3.9 ± 3.0% among the 53 animals (S1 Table). By the end of the six week period of sensitization to the aeroallergen, each cat had developed allergic asthma according to eosinophil counts elevated to an average of 54.9 ± 23.9% and exceeding 17% in all 53 cats (S1 Table).

### Preprocessing of NMR Spectra of EBC for Statistical Comparisons

In pursuit of metabolomics discrimination and biomarkers of early allergic asthma, 1D NMR spectra were acquired for the EBC specimens of each of the 53 before and after inducing the asthma. Overall concentrations of solutes in EBC samples varied over a 5-fold range. To remove the impact of this large variation in overall metabolite concentrations among the samples, the peak heights in the NMR spectra were normalized by PQN [31] (S1 Fig). Normalization is part of the spectral binning (untargeted) strategy [26] and enables statistical comparison of the *proportions* that the NMR peaks represent in the samples (in contrast to *absolute concentrations* of selected metabolites). PQN suppresses artifacts introduced by normalizing to the integral [31]. This improves the reliability of statistical comparisons of EBC specimens collected from patients with potential variations in exhaled volume, pulmonary gas exchange, and ambient humidity in the condensation chamber. The untargeted approach was also motivated by the biomarkers being unknown during the undertaking and development of the project. Since scaling methods influence statistical results [56], the effects of Pareto scaling (Eq 1) and *glog* transformation (Eq 2) [42] were compared. Without scaling, the bins with highest intensities (which tend to have higher variances) dominate the total variances (S2 Fig) and biased the statistics towards the tallest NMR peaks. Pareto scaling improved the situation but still resulted in a large range of variance among the bins (S2 Fig). The *glog* transformation gives greater weight to small variances that most likely result from small peak heights (S1 and S2 Figs). Preprocessing with PQN and *glog* transformation proved essential to discriminating asthma from health by enabling many spectral regions besides those of concentrated lactate to be considered by multivariate statistical analyses.

### Principal Component Analysis of the NMR Spectra

**Fig 1:**
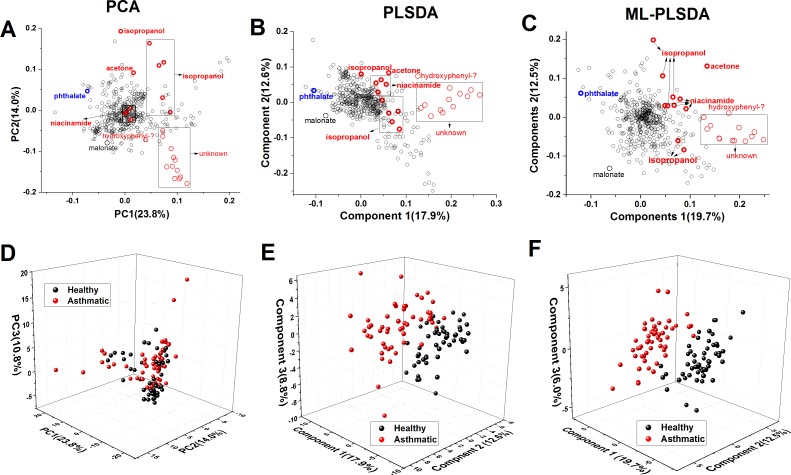
Comparison of loading and score plots generated from preprocessed NMR spectra of EBC from 53 cats before (black) and after induction of early allergic asthma (red), using multivariate statistics. Panels A and D show PCA results. Panels B and E plot PLS-DA results. Panels C and F report multi-level PLS-DA results. Components 1 and 2 are plotted in the loading plots (A–C). Components 1, 2, and 3 are plotted in the score plots (D–F). Biomarkers that increased in the experimental asthma are labeled in red. A marker that decreased in the experimental asthma is labeled in blue. A peak too weak to be useful is labeled in gray. Efforts to identify the markers are described below.

PCA was applied to the 106 preprocessed NMR spectra. The first two PCs account for about 38% of the total variance among the spectra (Fig 1A). The loading plot of PC1 and PC2 identifies many spectral bins with large variances among the 106 spectra. Metabolites represented by some of these spectral bins, identified by efforts described below, are labeled on the loading plots (Fig 1A–1C). The 3D plot of scores using the first three PCs does not adequately separate the healthy and asthmatic states of the cats (Fig 1D). This is due to unsupervised PCA placing high weights on the high variance of NMR peaks that are uncorrelated with the disease state of the cats.

### Partial Least Squares Improves Separation of Asthma from Health

Supervised PLS-DA methods were applied in a quest for better separation of asthmatic and healthy states of the cats and to recognize diagnostic spectral features. Five PLS components optimized the performance and reliability of the separation of the cats when healthy or asthmatic, as judged by the decrease of the root mean square error of the prediction (RMSEP) and the increase of the Q^2^ value (S3 Fig) from leave-one-out cross-validation. PLS-DA achieved much better separation than PCA in the score plot between cats before and after developing early allergic asthma (Fig 1D and 1E), presumably due to increased weights on variables that distinguish the health of the cats.

Multi-level PLS-DA was introduced to paired data sets to separate between-subject variation (e.g., due to genetic background) and within-subject variation (effect of “treatment”) in the data [35]. The crossover design of our data set (before and after induction of asthma) allowed us to test if multi-level PLS-DA could improve performance. After removing the between-subject variation, the loading plot is better dispersed than that of PLS-DA (Fig 1B and 1C). The score plot from multi-level PLS-DA better separates the groups, provided that the PQN type of normalization has been used (Fig 1F).

The spectra were preprocessed further using orthogonal signal correction (OSC) that removed two OSC components. This filtered out variances between spectra unrelated to separation between health and asthma in subsequent PLS-DA and multi-level PLS-DA calculations. Plots of RMSEP and Q^2^ generated by leave-one-out cross-validation showed that OSC-PLS-DA and multi-level PLS-DA perform clearly better in predictions than PLS-DA (S3 Fig). Multi-level OSC-PLS-DA performed the best of all by these criteria (S3 Fig). OSC-PLS-DA and multi-level PLS-DA reduced the RMSEP and increased Q^2^ more significantly than did PLS-DA when using the same number of components (S3 Fig). The first three to four components are enough to optimize the performance OSC-PLS-DA, which is a simplifying advantage over PLS-DA (S3 Fig). Two to three components suffice for multi-level OSC-PLS-DA to offer superior predictive power (S3 Fig).

**Fig 2:**
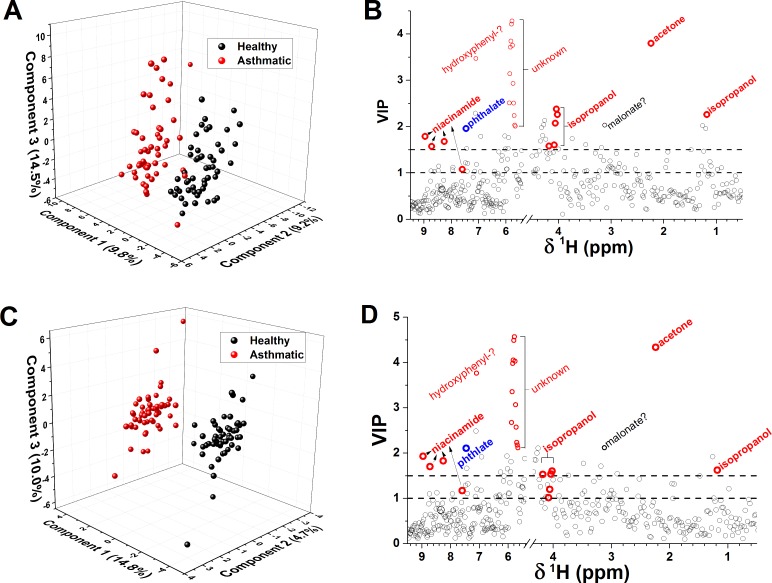
Orthogonal signal-corrected statistical results for the preprocessed spectra of EBC from 53 cats before and after induction of allergic asthma. Panels (A,B) show results from OSC-PLS-DA. Panels (C,D) show results from multi-level OSC-PLS-DA. (A,C) Scores are plotted, using the three largest principal components, for specimens collected before (black) and after induction of asthma (red). (B,D) The variable importance in projection (VIP) plots suggest the spectral features that most distinguish the experimental asthma from the health of the cats. As VIP values rise above 1.0, they imply increasing diagnostic significance [54]. Biomarkers increased by feline asthma are labeled red. A marker decreased by the experimental asthma is labeled blue. The weakness of the tentatively assigned NMR peak of malonate (labeled in gray) diminishes its diagnostic value below that implied by the VIP values.

The score plot from OSC-PLS-DA using the first three components shows group separation (Fig 2A) that is much improved over PCA and PLS-DA (Fig 1D and 1E). In the 2D score plot form OSC-PLS-DA, around three spectra of EBC from asthma are not distinguished from health (Fig 2A). Multi-level OSC-PLS-DA fully resolves the specimens from heath and asthma in the score plot (Fig 2C), and needs only two components to do so (S4 Fig). In order to test the prediction power of the model, two-thirds of the 106 samples were randomly selected as the training set, and the remaining third was used as the test set. Monte Carlo cross-validation suggests that the OSC-PLS-DA model with four components has very good predictive power with Q^2^ = 0.70, contrasting the negative control of random permutation testing with Q^2^ = -0.07 (Table 1). Cross-validation suggests even better predictive power from the multi-level OSC-PLS-DA model with three components where Q^2^ = 0.84 and the randomly permuted negative control has Q^2^ = -0.15 (Table 1). The prediction for the training set (a third of the specimens) using the OSC-PLS-DA model with four components has sensitivity of 94.1% and specificity of 94.1% (Table 1). The multi-level OSC-PLS-DA separation of the specimens into healthy and asthmatic classes is annotated with the names of the cats on a 2D score plot in S4 Fig. The prediction from multi-level OSC-PLS-DA model using three components has sensitivity of 94.1% and specificity of 100% (Table 1).

**Table 1:** Sensitivity, specificity, and testing of predictions from the orthogonal signal-corrected models.

	OSC-PLSDA	ML-OSC-PLSDA
*Prediction (2/3 training*, *1/3 testing)*		
Sensitivity	94.1%	94.1%
Specificity	94.1%	100%
*Q*^*2*^ *of Model Testing*		
Cross-Validation	0.698 ± 0.042	0.844 ± 0.018
Permuted Model (random)	-0.068 ± 0.199	-0.151 ± 0.205
*P*-value	1.25e^-62^	4.25e^-71^
*RMSEP of Model Testing*		
Cross-Validation	0.345 ± 0.042	0.248 ± 0.048
Permuted Model (random)	0.607 ± 0.060	0.602 ± 0.053
*P*-value	3.03e^-81^	3.04e^-116^

### NMR Identification of Metabolites in EBC from Cats

**Fig 3:**
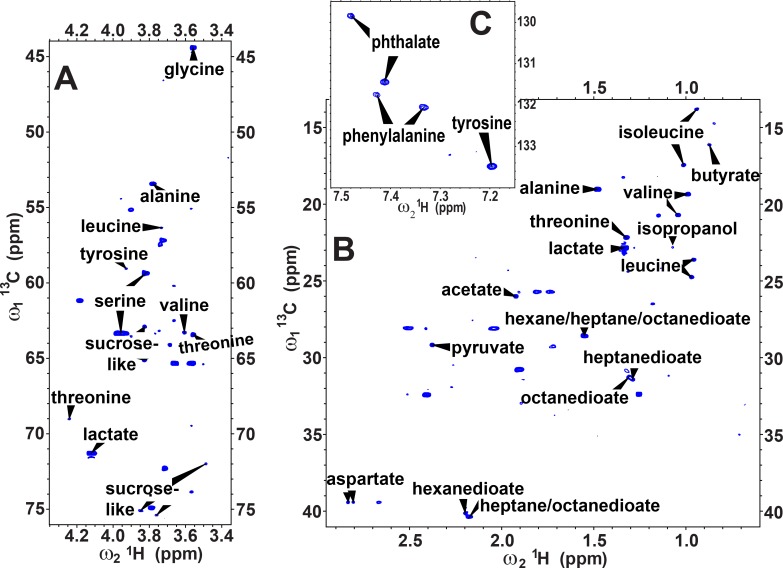
Multiple carboxylic acids are exhaled by cats. Regions of a natural abundance ^13^C HSQC NMR spectrum of a comparatively concentrated EBC specimen from a healthy cat are plotted with assignments for (A) the methine (-CH) region, (B) the methylene and methyl regions, and (C) the aromatic region. The sensitivity-enhanced HSQC was acquired at 25°C for 17.5 h at 800 MHz with a cryoprobe. Apodization with a squared sine bell introduced enough line broadening in the ^1^H dimension to mask multiplet splittings.

Independently from the untargeted statistical process of discriminating asthma from heath, we sought to identify more of the metabolites in feline EBC using two strategies. The more informative strategy proved to be a ^13^C HSQC spectrum of a relatively concentrated EBC sample from a healthy cat (Fig 3). Compounds present in the natural abundance ^13^C HSQC spectrum were analyzed using the COLMAR ^13^C HSQC database and server [51] that matches the spectrum against the MMCD and HMDB databases of NMR spectra of 555 compounds. Additional matching was done manually against reference NMR spectra available in HMDB [48, 49] and MMCD [50] databases. These efforts identified the amino acids alanine, aspartic acid, glycine, isoleucine, leucine, phenylalanine, serine, threonine, tyrosine, and valine (Fig 3). The ^13^C HSQC identified additional metabolically significant carboxylic acids of acetate, lactate, and pyruvate (Fig 3A and 3B). Also present are the dicarboxylic acids phthalate, hexanedioic acid (adipate) and either or both of heptanedioic acid (pimelate) and octanedioic acid (suberate), for which the NMR peaks overlap. Weak peaks for most but not all of the groups of sucrose or a related sugar are also observed (Fig 3A). An anomeric proton doublet at 5.41 ppm in 1D spectra is consistent with the presence of a sucrose-like molecule. A weak peak matching trimethylamine is also observed in the ^13^C HSQC (not shown). Overall, carboxylic acids predominate in NMR spectra of feline EBC.

**Fig 4:**
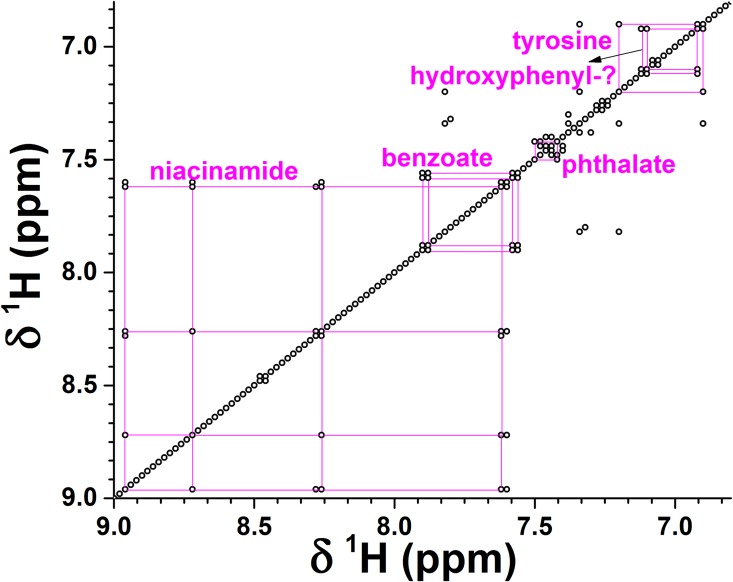
Covalent connectivity of metabolites in feline EBC is suggested by co-variation (STOCSY) among the 106 preprocessed NMR spectra. The aromatic region is plotted. Off-diagonal spots represent correlations (r ≥ 0.7) of two chemical groups across the spectra, suggesting that they belong to the same metabolite or pathway.

The second strategy to gain more information to identify and confirm molecules was to ascertain co-variation among the 106 preprocessed 1D NMR spectra and to plot the co-variation as a correlation map known as STOCSY [52] (Fig 4). Two or more co-varying NMR peaks lying away from the diagonal constitute a ‘spin system’ represented by spots along a row or column (Fig 4 and S5 Fig), which better define the identity of a molecule than a single peak can. Comparison of the positions of the correlations in the STOCSY against the database of TOCSY spectra at the COLMAR server [57] identified niacinamide, benzoate, and another aromatic metabolite that probably contains a hydroxyphenyl moiety (Fig 4). The STOCSY also confirmed phthalate and tyrosine. The aliphatic region of the STOCSY and comparison of spectra of EBC from cats with the HMDB database identifies isopropanol, propionate, and butyrate (S5 Fig). (The latter’s potential alternative assignment as methyl butanoate appears unlikely due to the absence from the STOCSY of a correlation to the methyl peak expected in the range of 3.35 to 3.65 ppm. Nor is there a correlation for isobutyrate.) The STOCSY confirms the assignments of lactate and hexanedioate (S5 Fig) based on the ^13^C HSQC (Fig 3A). The butyrate is confirmed by TOCSY spectra and multiplet line shapes in 1D NMR spectra. The propionate and isopropanol are also confirmed by 1D spectra. A number of spin systems evident in the STOCSY (S5 Fig) failed to match the online databases, suggesting the absence from the databases of multiple compounds present in EBC.

### Metabolites and Pathways Perturbed by Allergic Asthma

**Fig 5:**
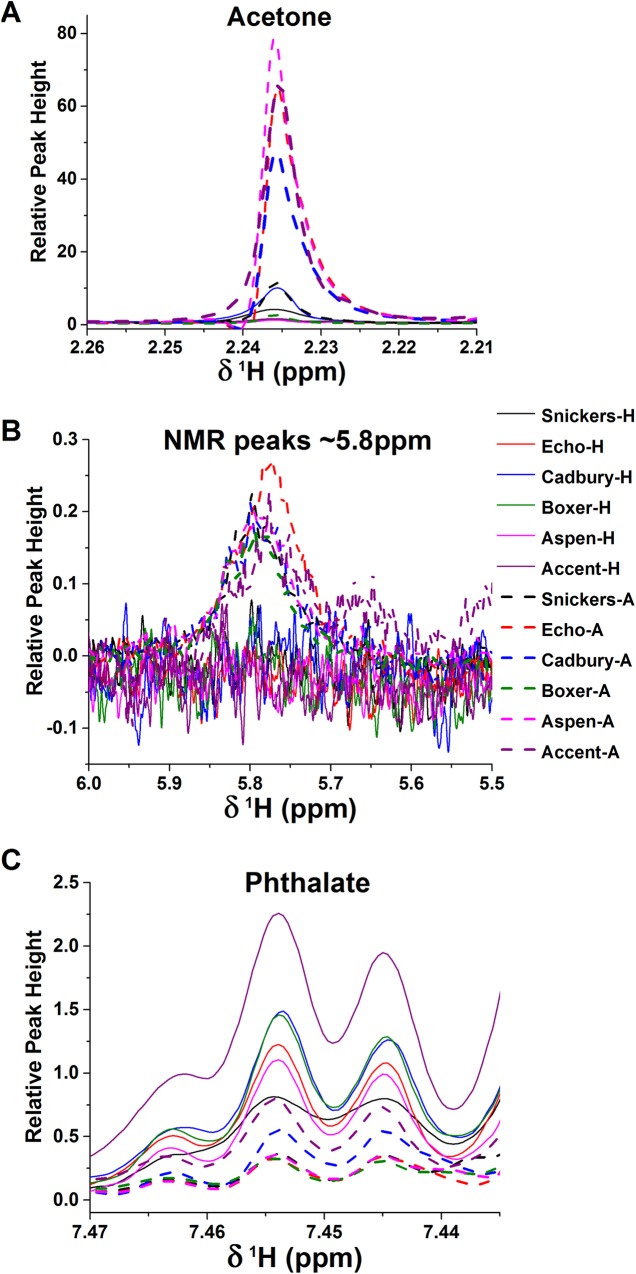
Changes in the NMR peaks of three biomarkers in several cats before (solid lines) and after development of allergic asthma (dashed lines). The spectra shown are PQN-normalized. The cats providing these EBC samples are named at right. (A) Acetone is increased in 74% of cats soon after developing allergic asthma, suggesting a shift in central energy metabolism. (B) The unidentified collection of broad, overlapped peaks that increased in 70% of the cats with experimental asthma is plotted. (C) The doublet NMR peak of phthalate is often decreased in the early allergic asthma. The legend names the cats sampled, with a suffix of H denoting health and a suffix of A denoting asthma.

The VIP plots from OSC-PLS-DA and multi-level OSC-PLS-DA suggest the spectral features that best distinguish the EBC of cats in states of health and early allergic asthma. The trends in the six best markers in the VIP plots were manually inspected in the 106 PQN-normalized NMR spectra (Table 2). The highest VIP score (Fig 2B and 2D) suggests acetone to be the best biomarker identified. Acetone in EBC is clearly elevated by early allergic asthma in 74% of the cats, in whom acetone was scarcely detectable during health (Table 2, Fig 5A). The unidentified metabolites giving rise to broad, overlapped peaks near 5.8 ppm are elevated by early allergic asthma in 70% of the cats (Fig 5B). The unidentified, probably hydroxyphenyl-containing aromatic metabolite is increased by the experimental asthma in 62% of the cats (Table 2). Isopropanol is increased in 51% of the cats when asthmatic, and niacinamide in 43% of them (Table 2). Leucine levels are not affected by asthma in 73% of the cats, rendering it unreliable as a marker despite VIP scores > 1. Decreased phthalate upon onset of experimental allergic asthma in 60% of the cats (Table 2, Fig 5C) suggests its diagnostic value, despite its origins in environmental sources.

**Table 2:** Biomarkers of early allergic asthma in cats, ranked in descending order of diagnostic value. The relative sizes of the NMR peaks were compared across the 106 probabilistic quotient normalized spectra.

Biomarker	Proportion of the 53 cats by relative level of biomarker		
	Increased in asthma	Decreased in asthma	Unchanged in asthma
**Acetone**	73.6%	17%	9.4%
**NMR peaks near 5.8 ppm**	69.8%	11.3%	18.9%
**Hydroxyphenyl-containing**	62.3%	1.9%	35.8%
**Phthalate**	30.2%	60.4%	9.4%
**Niacinamide**	43.4%	9.4%	47.2%
**Isopropanol**	50.7%	39.6%	9.4%

The primary marker of acetone and secondary markers of isopropanol and niacinamide were submitted to metabolic pathway analysis by MetaboAnalyst 3.0 [55, 58]. A *P*-value of 0.05 suggests that nicotinate and nicotinamide (niacinamide) metabolism could be perturbed in the asthmatic animals that exhale more niacinamide. NAD^+^, NADP^+^, nicotinamide ribotide, and nicotinamide riboside each are precursors to nicotinamide. The frequent elevation of acetone in early allergic asthma in cats implicates synthesis and degradation of ketone bodies with *P*-value of 0.007. Inclusion of isopropanol in the pathway analysis implies perturbation of propanoate metabolism by the experimental asthma, with the confidence of a *P*-value of 0.0006. Acetone accumulates in the majority of cats with allergic asthma presumably because of active synthesis of ketone bodies. This can be regarded as belonging to a larger set of pathways of propanoate metabolism. Isopropanol, which appears elevated in half of the cats with experimental asthma, is an immediate precursor to acetone. Acetoacetate is the better known precursor of acetone and lies on the pathway from acetyl-CoA, which is central in metabolism.

## Discussion

Novel noninvasive means for diagnosing asthma and monitoring its management present a significant need and opportunity in both pediatric care [15, 18, 19, 59] and veterinary care of pet cats [16]. Earlier studies reported statistically promising diagnostic potential for human asthma by collecting EBC and assaying it by NMR for asthma in both children [18] and adults [23], but did not identify biomarkers. The metabolomics results in the feline asthma model bolster these evidences for EBC being a noninvasively accessible fluid that is measurable by NMR for diagnostic value for asthma. Increases of acetone, a group of overlapped peaks near 5.8 ppm, and a hydroxyphenyl-like metabolite, as well as decreases of phthalate, have emerged as the best NMR-detectable markers in EBC of early allergic asthma in cats (Figs 2 and 5 and Table 2). Increases of isopropanol and niacinamide in EBC are confirmatory of allergic asthma in part of the cats. These NMR-detected observations complement the volatile organics detected by GC-MS [25, 60] or electronic nose [61].

### Ketosis in Allergic Asthma

Clinically relevant ketones in cats produced during states of decreased glucose utilization or negative energy balance include acetone, β-hydroxybutyrate, and acetoacetate. In 74% of the cats of this study, exhaled acetone was increased with induction of allergic asthma, implying increased ketogenesis. The acetone may have been produced locally by microbiota in the lung or emanated from the circulation of these cats. Acetone is one of the serum metabolites that was found to be related to the severity of eosinophilic asthma and airflow limitation in adult humans [62]. In cats, increased serum β-hydroxybutyrate, is characteristic of diabetes [63], diabetic ketoacidosis, hepatic lipidosis, and less frequently other conditions such as chronic kidney disease and hyperthyroidism in which fat is used as an energy source [64, 65]. Ketonemia in cats is typically associated with a shift from glucose utilization to β-oxidation of fatty acids and negative energy balance [64, 66]. No evidence of negative energy balance was noted in the 53 cats of this study however.

Similarly in humans, increases of acetone in serum accompanied decreases of % forced expiratory volume in 1 s (FEV_1_%), an important manifestation of asthma [62, 67]. The lower FEV_1_% was also correlated with much increased very low and low density lipoprotein (VLDL/LDL), which is strongly suggestive of altered lipid metabolism [62, 67]. The authors suggested the increases of acetone and histamine release result from the elevation of VLDL/LDL [67]. This could be related to the increased exhalation of volatile organic compounds in asthma, which has been attributed to increased peroxidation of lipids [25, 60].

### Other Metabolic Biomarkers of Asthma

The decrease of glucose and increase of lactate in serum from adult humans with asthma, detected by NMR-based metabolomics, may be suggestive of hypoxia [67]. Those changes could be consistent with the increase of exhaled acetone and apparent ketogenesis in asthmatic cats. At the early stage of feline allergic asthma, the changes in lactate levels were, however, highly heterogeneous and without clear trend (not shown). This may be attributable to variability in the recent exercise and nutrition of the cats. An NMR-detected study of EBC from adults suggested mild asthma to decrease short-chain fatty acids, valine, formate, hippurate and urocanic acid, as well as to increase proline, propionate, isobutyrate, and phenylalanine [24], Such changes cannot, however, be corroborated in cat EBC upon onset of experimental asthma. The human study normalized the EBC spectra to their spectral area [24], a common practice that can introduce artifacts of some metabolites *appearing* to decrease [30]. The study of EBC of adult humans made no mention of an increase of acetone by asthma. These differences in prospective biomarkers of asthma suggest the need for caution and limits to generalizing across very different cohorts of asthma patients.

Why was phthalate observed in this feline study? Phthalates are found in many consumer products. Humans consume phthalate esters introduced to the diet by the processing of foods, especially food packaging films and meats, which contain di-2-ethylhexylphthalate [68–74]. The dry kibble diet of the cats was highly processed and contained animal fats, i.e. likely sources of phthalate esters by analogy with the contamination documented in human diets. Perhaps exhalation could be a route of elimination of phthalates that was impaired by the experimental asthma in the cats.

The most serious technical impediment to use of EBC from cats is the inherent large variability, which appears unrelated to the pulmonary disease state. This stymied PCA from distinguishing early experimental asthma from health. Fortunately, probabilistic quotient normalization [31] enabled successful discrimination using supervised statistical methods based on PLS-DA. For the best diagnostic separations, multi-level PLS-DA or preprocessing with orthogonal signal correction suppressed much variability that interfered in diagnostic classification. With OSC-PLS-DA, the predictive model was simplified to only three to four components and attained predictive power of 94% sensitivity and 94% specificity (Table 1). Multi-level suppression of between-subject variability [35] combined in novel fashion with orthogonal signal correction (Fig 2C) notably increased the specificity in predicting asthma to 100% (Table 1). Consequently, the combination of OSC and multi-level enhancements to PLS-DA appears promising for comparative metabolomic and clinical research studies where specimens can be assayed from each subject before and after a treatment or change in clinical status.

## Conclusions

After preprocessing of NMR spectra with probabilistic quotient normalization and *glog* transformation, OSC-PLS-DA and multi-level PLS-DA overcame confounding variability among EBC samples to distinguish asthma from health noninvasively in a cohort of research cats. The predictive ability of the OSC-PLS-DA model is promising as both the sensitivity and specificity are 94%. The promising biomarkers of allergic asthma in cats to emerge in their EBC are increases in acetone, unidentified metabolite(s) with broad NMR peaks near 5.8 ppm, and an aromatic compound probably containing a hydroxyphenyl group. Also promising is the decrease of phthalate in 60% of the cats with asthma. The noninvasive, untargeted approach utilizing properly preprocessed NMR spectra of EBC interpreted with OSC-PLS-DA appears worth further evaluation for translational research. Reliable differential diagnosis of early asthma in children is especially needed.

## Supporting Information

S1 Fig Effects of preprocessing steps on spectra of EBC collected before and after induction of early allergic asthma in a cat. (A) The original spectra are plotted. (B) The spectra are plotted after probabilistic quotient normalization (PQN). (C) The PQN-normalized spectra were segmented into bins of 0.02 ppm width and plotted. (D) The binned spectra were scaled by *glog* transformation.
(TIF)

S2 Fig Evaluation of scaling methods for improving prospects for multivariate statistics by distributing variances more equitably among peaks of the NMR spectra. (A) The variance of each bin is plotted against the ranked mean of the data before (black) and after either Pareto scaling (red) or *glog* transformation (blue). *Glog* transformation redistributes the variance among the NMR spectra of feline EBC more widely and evenly than does Pareto scaling. (B) Calibration of the key λ parameter of *glog* transformation (Eq 2) is shown. λ was optimized using the Maximum likelihood criterion and the Nelder-Mead minimization algorithm in MATLAB as previously described by Parsons et al. (2007). SSE refers to the sum of the squared errors. The optimized λ = 7.183X10^12^ that minimizes SSE was used in spectral preprocessing.
(TIF)

S3 Fig **RMSEP (A) and Q**^**2**^ **plots (B) from leave-one-out cross-validation** from PLS-DA (black squares), OSC-PLS-DA (blue triangles), ML-PLS-DA (red circles), and ML-OSC-PLSDA (pink triangles). The orthogonal signal correction (OSC) decreases the number of components needed.
(TIF)

S4 Fig Score plot from multi-level OSC-PLS-DA that includes names of the 53 cats. This corresponds to Fig 2C, but plots only components 1 and 2. Health is symbolized by black squares and a suffix of H. Asthma is symbolized by red circles and a suffix of A.
(TIF)

S5 Fig Aliphatic region of the STOCSY correlation map among EBC spectra. Spin systems of some recognizable metabolites are labeled.
(TIF)

S1 Table Eosinophil counts of 53 cats before and after induction of experimental asthma. (DOCX)

## Abbreviations

BALF: bronchoalveolar lavage fluid
COLMAR: Complex Mixture Analysis by NMR
EBC: exhaled breath condensate
GC-MS: gas chromatography–mass spectrometry
HMDB: human metabolomics database
HSQC: heteronuclear single quantum coherence
MMCD: Madison-Qingdao Metabolomics Consortium Database
OSC: orthogonal signal correction
PC: principal component
PCA: principal component analysis
PLS-DA: partial least squares with discriminant analysis
OSC-PLS-DA: orthogonal signal correction partial least squares with discriminant analysis
PQN: probabilistic quotient normalization
RMSEP: root-mean-square-error-of-prediction
STOCSY: statistical total correlation spectroscopy
VIP: variable importance in projection
