# Supplementary figures and images for: Noninvasive Recognition and Biomarkers of Early Allergic Asthma in Cats Using Multivariate Statistical Analysis of NMR Spectra of Exhaled Breath Condensate
Source: PLoS One. 2016 Oct 20;11(10):e0164394. doi: 10.1371/journal.pone.0164394 (PMC5072706; doi:10.1371/journal.pone.0164394)

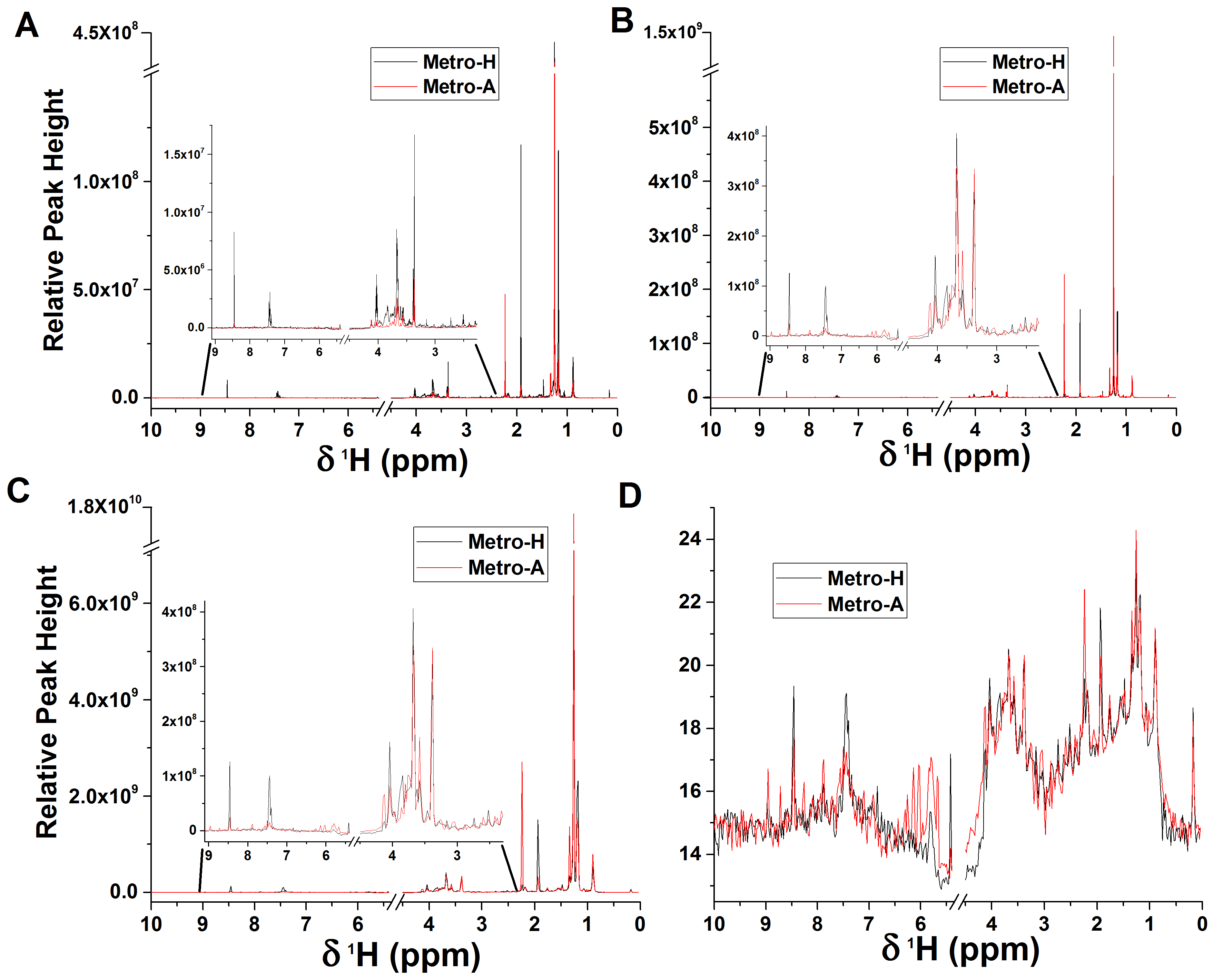

Supplement: S1 Fig — (A) The original spectra are plotted. (B) The spectra are plotted after probabilistic quotient normalization (PQN). (C) The PQN-normalized spectra were segmented into bins of 0.02 ppm width and plotted. (D) The binned spectra were scaled by glog transformation. (TIF) [file pone.0164394.s001.tif]

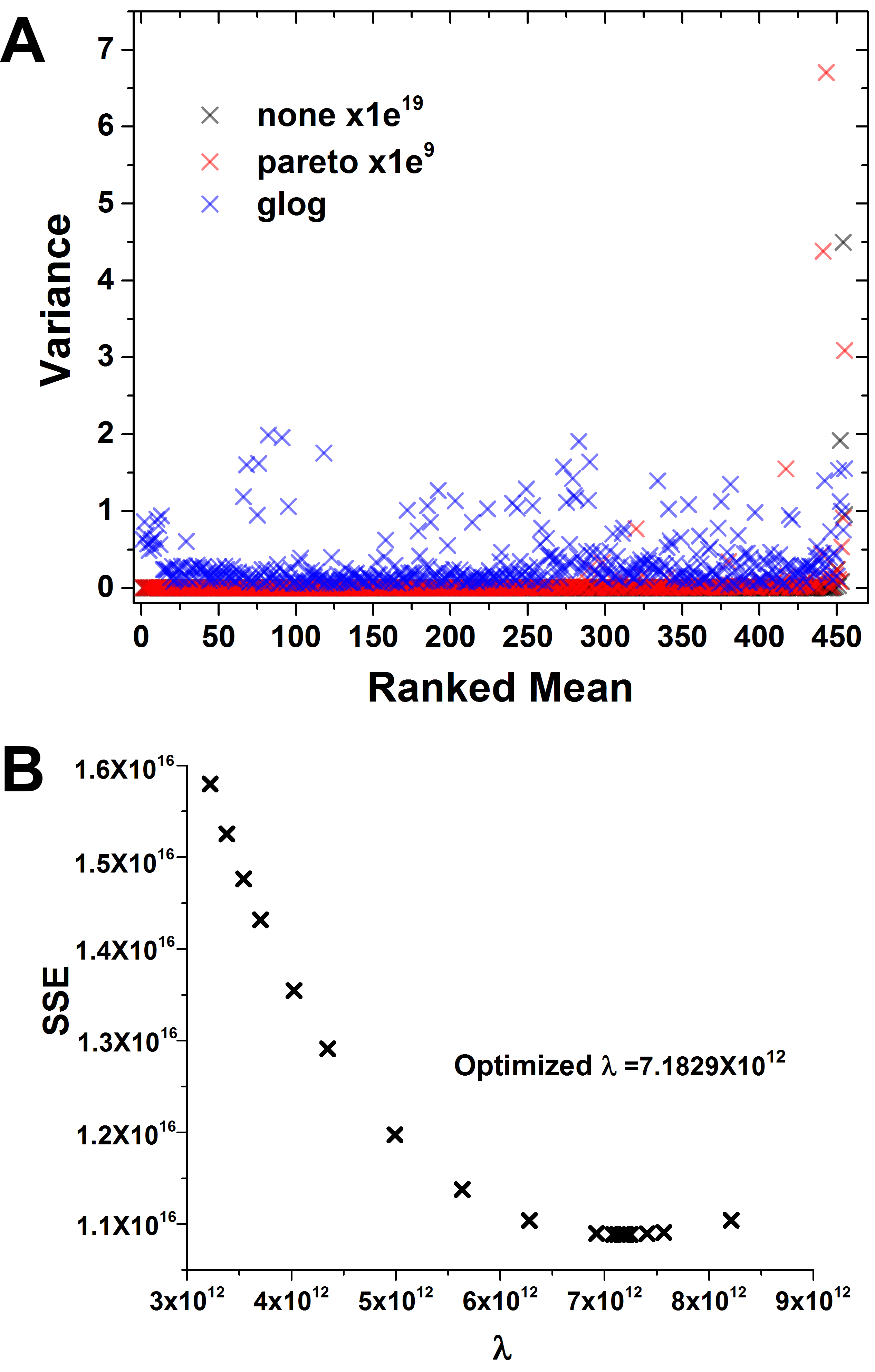

Supplement: S2 Fig — (A) The variance of each bin is plotted against the ranked mean of the data before (black) and after either Pareto scaling (red) or glog transformation (blue). Glog transformation redistributes the variance among the NMR spectra of feline EBC more widely and evenly than does Pareto scaling. (B) Calibration of the key λ parameter of glog transformation (Eq 2) is shown. λ was optimized using the Maximum likelihood criterion and the Nelder-Mead minimization algorithm in MATLAB as previously described by Parsons et al. (2007). SSE refers to the sum of the squared errors. The optimized λ = 7.183X1012 that minimizes SSE was used in spectral preprocessing. (TIF) [file pone.0164394.s002.tif]

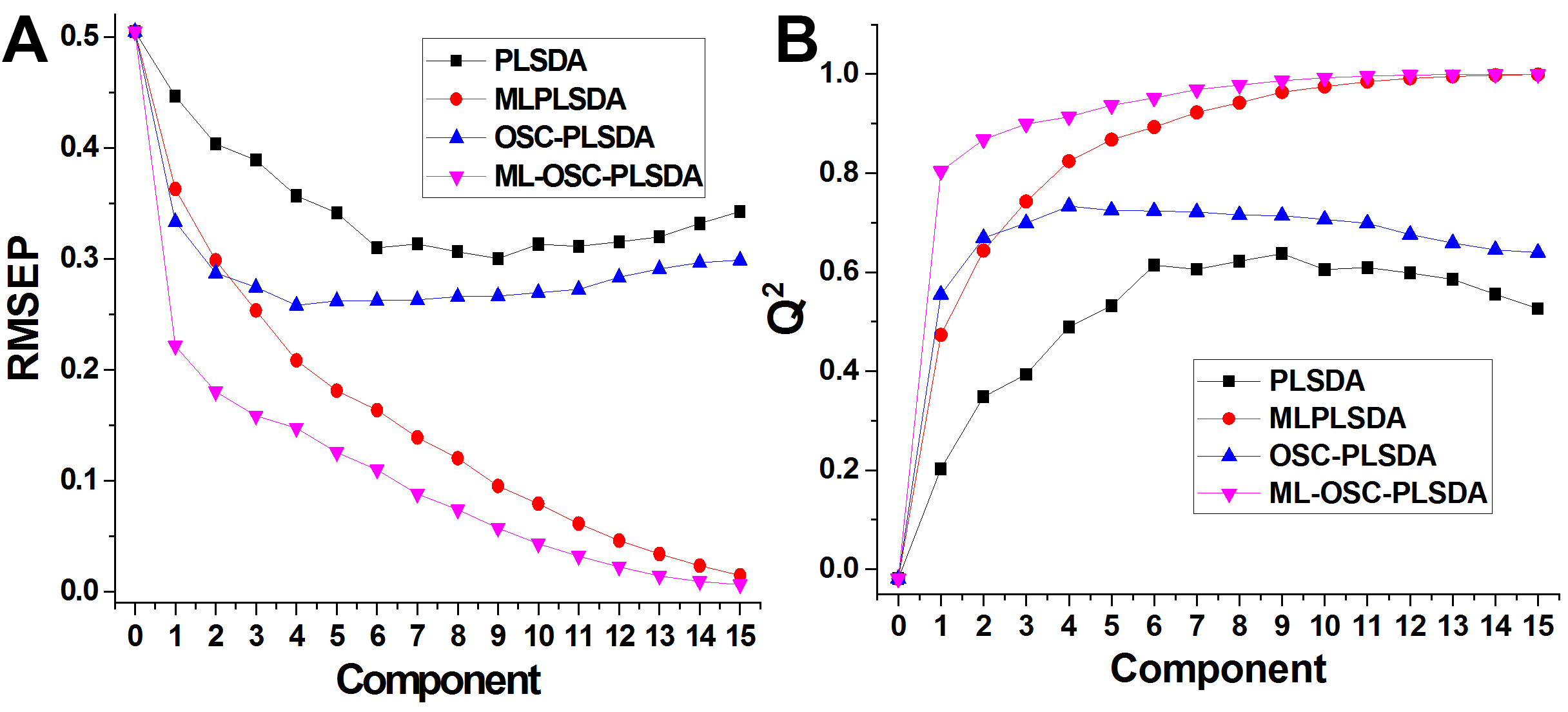

Supplement: S3 Fig — RMSEP (A) and Q2 plots (B) from leave-one-out cross-validation from PLS-DA (black squares), OSC-PLS-DA (blue triangles), ML-PLS-DA (red circles), and ML-OSC-PLSDA (pink triangles). The orthogonal signal correction (OSC) decreases the number of components needed. (TIF) [file pone.0164394.s003.tif]

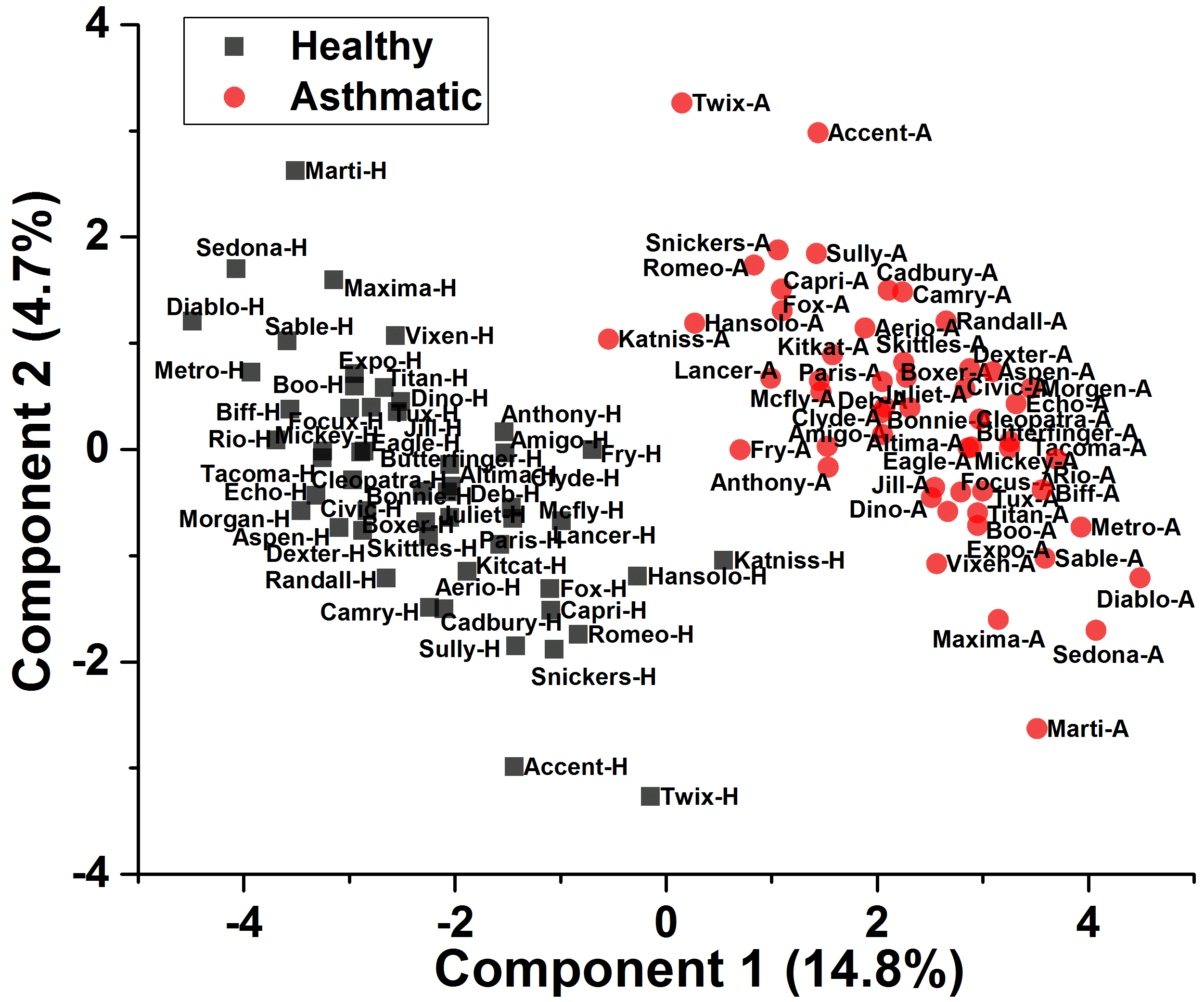

Supplement: S4 Fig — This corresponds to Fig 2C, but plots only components 1 and 2. Health is symbolized by black squares and a suffix of H. Asthma is symbolized by red circles and a suffix of A. (TIF) [file pone.0164394.s004.tif]

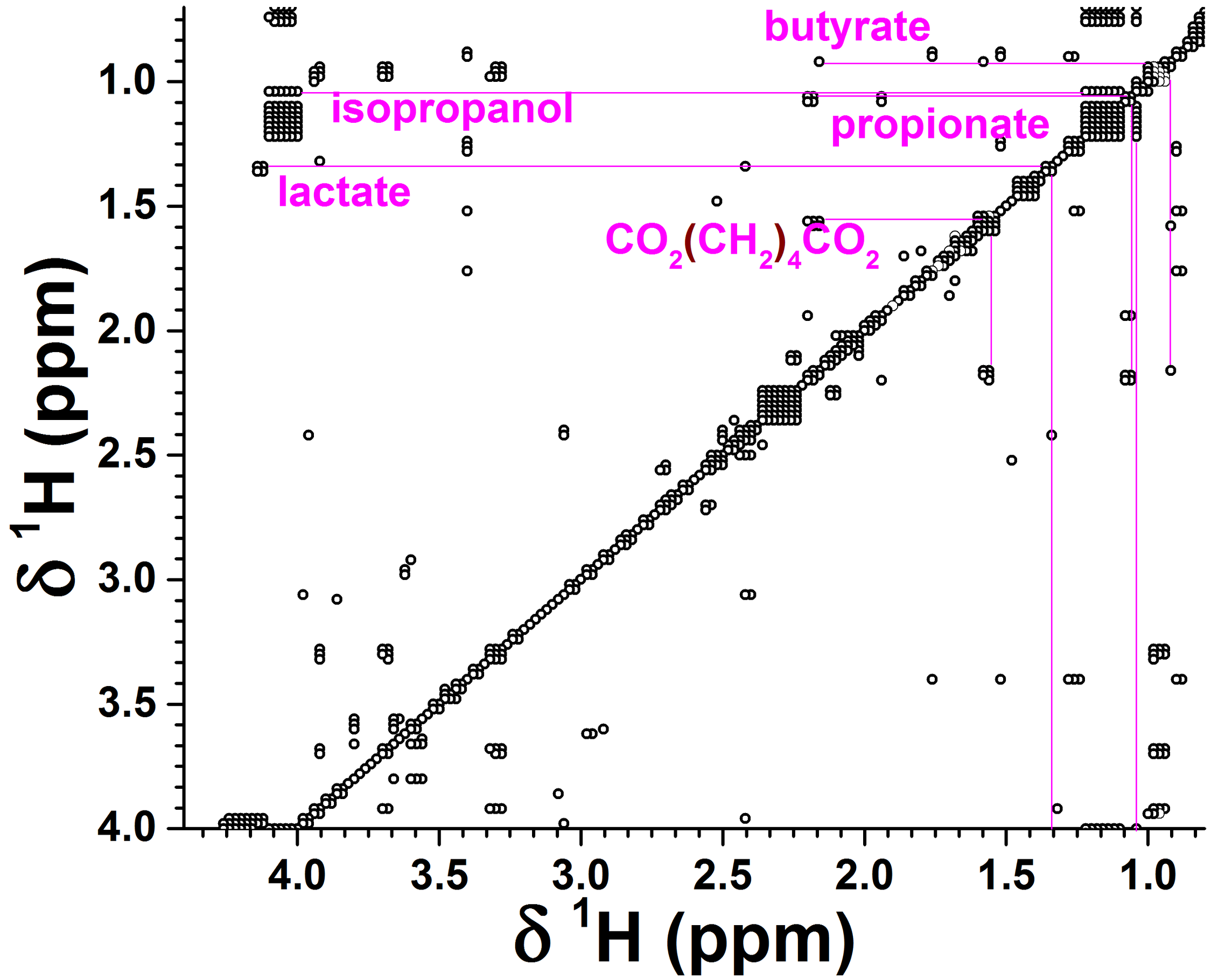

Supplement: S5 Fig — Spin systems of some recognizable metabolites are labeled. (TIF) [file pone.0164394.s005.tif]
