# Supplementary material for: Noninvasive Recognition and Biomarkers of Early Allergic Asthma in Cats Using Multivariate Statistical Analysis of NMR Spectra of Exhaled Breath Condensate
Source: PLoS One. 2016 Oct 20;11(10):e0164394. doi: 10.1371/journal.pone.0164394 (PMC5072706; doi:10.1371/journal.pone.0164394)
Supplement: S1 Table — (DOCX) [file pone.0164394.s006.docx]

**S1 Table**. **Eosinophil counts of 53 cats before and after induction of experimental asthma**.

| Cat Name | % eosinophils | | | Cat Name | % eosinophils | | Cat Name | % eosinophils | |
| --- | --- | --- | --- | --- | --- | --- | --- | --- | --- |
|  | **Health** | | **Asthma** |  | **Health** | **Asthma** |  | **Health** | **Asthma** |
| Vixen^b^ | 5 | 22 | | **Marti** ^b^ | 0 | 57 | **Cleopatra**^b^ | 6 | 68 |
| Twix | 2 | 61 | | **Lancer** | 5 | 85 | **Civic** | 10 | 39 |
| Tux | 4 | 32 | | **Juliet** ^b^ | 4.5 | 76 | **Capri** | 8 | 49 |
| Titan | 0 | 26 | | **Jill** ^b^ | 4 | 58 | **Camry** | 9 | 41 |
| Tacoma | 5 | 26 | | **Kitcat** ^b^ | 4 | 74 | **Cadbury** | 2 | 81 |
| Sully | 0 | 17 | | **Katniss**^b^ | 1 | 85 | **Butterfinger** | 1 | 71 |
| Snickers^b^ | 3 | 74 | | **Fry**^b^ | 0 | 85 | **Boxer** | 5 | 79 |
| Skittles^b^ | 4 | 70 | | **Fox** | 29^a^ | 89 | **Boo**^b^ | 4 | 72 |
| Sedona | 4 | 23 | | **Focus** | 2 | 42 | **Bonnie**^b^ | 5 | 64 |
| Sable | 3 | 18 | | **Hansolo** | 0 | 70 | **Biff** | 6 | 29 |
| Romeo | 3 | 74 | | **Expo** | 34^a^ | 77 | **Aspen** | 1 | 84 |
| Rio | 21 ^a^ | 42 | | **Echo** | 4 | 87 | **Anthony** | 2.5 | 40 |
| Randall | 1 | 81 | | **Eagle** | 7 | 94 | **Amigo** | 4 | 75 |
| Morgan^b^ | 0 | 46 | | **Dino** | 2 | 69 | **Altima** | 13 | 30 |
| Mickey | 6.5 | 49 | | **Diablo** | 10 | 24 | **Aerio** | 3 | 24 |
| Metro | 9 | 44 | | **Dexter** | 1 | 35 | **Accent** | 4 | 17 |
| Mcfly | 4 | 69 | | **Deb**^b^ | 3 | 23 | mean | 3.9 | 54.9 |
| Maxima | 0 | 34 | | **Clyde** | 5.5 | 52 | std. dev. | 3.0 | 23.9 |

^a^ Despite high eosinophils, the cat lacked symptoms of asthma. Intradermal skin tests were negative. (Parasites are a more likely source of high eosinophils in this cat less than a year old). The cat responded robustly to allergen sensitization with large increases in eosinophils.

^b^ gender is female
